# Senescence Rejuvenation through Reduction in Mitochondrial Reactive Oxygen Species Generation by *Polygonum cuspidatum* Extract: In Vitro Evidence

**DOI:** 10.3390/antiox13091110

**Published:** 2024-09-14

**Authors:** Jee Hee Yoon, Ye Hyang Kim, Eun Young Jeong, Yun Haeng Lee, Youngjoo Byun, Song Seok Shin, Joon Tae Park

**Affiliations:** 1 Division of Life Sciences, College of Life Sciences and Bioengineering, Incheon National University, Incheon 22012, Republic of Korea; yoojn0905@inu.ac.kr (J.H.Y.); yh.lee@inu.ac.kr (Y.H.L.); 2 Hyundai Bioland Co., Ltd., 22, Osongsaengmyeong 2-ro, Osong-eup, Heungdeok-gu, Cheongju-si 28162, Republic of Korea; dpgid27@hyundaibioland.co.kr (Y.H.K.); eyjeong99@hyundaibioland.co.kr (E.Y.J.); 3 College of Pharmacy, Korea University, Sejong 30019, Republic of Korea; yjbyun1@korea.ac.kr; 4 Convergence Research Center for Insect Vectors, Incheon National University, Incheon 22012, Republic of Korea

**Keywords:** reactive oxygen species (ROS), oxidative stress, senescence rejuvenation, *Polygonum cuspidatum*, skin aging

## Abstract

Oxidative stress caused by reactive oxygen species (ROS) is one of the major causes of senescence. Strategies to reduce ROS are known to be important factors in reversing senescence, but effective strategies have not been found. In this study, we screened substances commonly used as cosmetic additives to find substances with antioxidant effects. *Polygonum cuspidatum* (*P. cuspidatum*) extract significantly reduced ROS levels in senescent cells. A novel mechanism was discovered in which *P. cuspidatum* extract reduced ROS, a byproduct of inefficient oxidative phosphorylation (OXPHOS), by increasing OXPHOS efficiency. The reduction in ROS by *P. cuspidatum* extract restored senescence-associated phenotypes and enhanced skin protection. Then, we identified polydatin as the active ingredient of *P. cuspidatum* extract that exhibited antioxidant effects. Polydatin, which contains stilbenoid polyphenols that act as singlet oxygen scavengers through redox reactions, increased OXPHOS efficiency and subsequently restored senescence-associated phenotypes. In summary, our data confirmed the effects of *P. cuspidatum* extract on senescence rejuvenation and skin protection through ROS reduction. This novel finding may be used as a treatment in senescence rejuvenation in clinical and cosmetic fields.

## 1. Introduction

The skin is composed of three layers (the epidermis, dermis, and subcutaneous fat) and is an epithelial tissue that covers our body [1]. The skin acts as a barrier to the environment, protects against microorganisms, and maintains fluid and temperature. Skin aging is a process in which skin quality deteriorates with age [2]. Skin aging is indicated by changes in the function of cellular organelles, most notably mitochondrial degeneration [3]. As skin ages, defective mitochondria accumulate and undergo structural changes that significantly increase mitochondrial volume and size [4]. Defective mitochondria leak electrons from complex I and III in the electron transport complex (ETC) to generate ROS as byproducts [5]. In addition to producing ROS, defective mitochondria are the target of oxidative stress, which further increases mitochondrial ROS generation. Increase in oxidative stress by ROS worsens skin aging by inducing protein oxidation, lipid peroxidation, and chain scission of collagen and elastin [6]. Thus, strategies to reduce mitochondrial ROS generation might be effective as a therapeutic approach for skin aging [7,8].

*Polygonum cuspidatum* (*P. cuspidatum*) extract has been traditionally used as a medicine for various diseases in China, Japan, and Korea [9,10,11]. Traditionally, *P. cuspidatum* extract has been used as a folk remedy to treat patients suffering from indigestion and gastrointestinal disorders [12,13]. Recently, *P. cuspidatum* extract was found to have antiviral activity against coronavirus 2 by preventing the interaction between the viral spike protein and host–cell receptors [14]. It also showed remarkable antibacterial effects against *Escherichia coli*, *Streptococcus mutans*, and *Streptococcus sobrinus* [15]. In addition to its antiviral and antibacterial effects, *P. cuspidatum* extract has been used as a cosmetic ingredient. Specifically, *P. cuspidatum* extract was effective in skin whitening and improving blemishes [16,17,18]. However, the underlying mechanism and active ingredients by which *P. cuspidatum* extract exerts its effects have not yet been investigated. Therefore, understanding the mechanism of the skin-beautifying effects of *P. cuspidatum* extract and identifying its active ingredients will further expand the applications of *P. cuspidatum* extract as an anti-aging agent and cosmetic additive.

In this study, we found that *P. cuspidatum* extract is an antioxidant that significantly reduces ROS levels in senescent fibroblasts. A novel ROS-reducing mechanism by *P. cuspidatum* extract was identified, through which it restored senescence-associated phenotypes and skin barrier function. Furthermore, we identified which active components of *P. cuspidatum* extract played a key role in demonstrating these effects. Herein, we propose a novel ROS-reducing mechanism of *P. cuspidatum* extract to improve senescence-associated phenotypes and enhance skin protection.

## 2. Materials and Methods

### 2.1. Cell Culture

Human dermal fibroblasts (PCS-201-010; ATCC, Manassas, VA, USA), immortalized human keratinocytes (HaCaT; 300493; Cytion, Eppelheim, Germany), and normal human epidermal keratinocytes (HEKn; C0055C; Gibco, Grand Island, NY, USA) were used. Human dermal fibroblasts were maintained in Dulbecco’s modified Eagle’s medium (DMEM) supplemented with 10% fetal bovine serum (FBS; SH30919.03; Hyclone, Waltham, MA, USA) and 100 U/mL penicillin/100 μg/mL streptomycin (SV30079.01; Hyclone). HaCaT cells were maintained in DMEM supplemented with 10% FBS (SH30919.03; Hyclone), 2 mM L-glutamine (A2916801; Thermo Fisher Scientific, Waltham, MA, USA) and 100 U/mL penicillin/100 μg/mL streptomycin (SV30079.01; Hyclone). HEKn cells were maintained in EpiLife^®^ medium (M-EPICF-500; Thermo Fisher Scientific) supplemented with 1× human keratinocyte growth supplement (S001K; Thermo Fisher Scientific) and 10 μg/mL gentamicin/0.25 μg/mL amphotericin B (R01510; Thermo Fisher Scientific). Cells were cultivated in ambient air (20% O_2_) with 5% CO_2_ at 37 °C. The culture media was changed every four days. Using a Cedex HiRes Analyzer (05650216001; Roche, Basel, Switzerland), cell counts and viability were evaluated. The doubling time of human dermal fibroblasts was used to categorize them as either senescent or young, depending on whether it was 14 days or more or less than 2 days.

### 2.2. Preparation of P. cuspidatum Extract, Pyrroloquinoline Quinone (PQQ), and Caffeine

The roots of *P. cuspidatum* were mixed with distilled water in a volume ratio of 1:10 and heated at 60 °C for 5 h. The extract was filtered using a 5 μm filter and then using a 0.45 μm filter. The filtrate was completely concentrated using a vacuum evaporator and then dried using a vacuum dryer (OV-12; JEIOTECH, Daejon, Republic of Korea). *P. cuspidatum* extract was diluted to a concentration of 100 mg/mL using dimethyl sulfoxide (DMSO, D8418; Sigma, St. Louis, MO, USA). To make a concentration of 10 μg/mL *P. cuspidatum* extract, 1 μL of 100 mg/mL *P. cuspidatum* extract was added to 10 mL medium. DMSO control was used by diluting DMSO in the medium to a concentration of 0.01%. To make the DMSO (0.01%), 1 μL of DMSO was added to 10 mL medium. Pyrroloquinoline quinone (PQQ, D7783; Sigma) was diluted to a concentration of 100 mg/mL using DMSO. To make a concentration of 10 μg/mL PQQ, 1 μL of 100 mg/mL PQQ was added to 10 mL medium. Caffeine (C7731; Sigma) was diluted to a concentration of 100 mg/mL using DMSO. To make a concentration of 10 μg/mL caffeine, 1 μL of 100 mg/mL caffeine was added to 10 mL medium.

### 2.3. Flow Cytometric Analysis of Reactive Oxygen Species (ROS)

*P. cuspidatum* extract, pyrroloquinoline quinone (PQQ, 80198; Sigma), and caffeine (C0750; Sigma) were diluted to a final concentration of 10 µg/mL and treated to senescent fibroblasts for 12 days. DMSO control was used by diluting DMSO in the medium to a concentration of 0.01%. As a positive control, 100 µM resveratrol (76511; Sigma) was used. Then, cells were incubated for 30 min at 37 °C in a medium 30 µM DHR123 (10056-1; Biotium, Fremont, CA, USA) in order to quantify ROS. Then, cells were processed for flow cytometry analysis as previously explained [19].

### 2.4. Cellular Proliferation Assay

Senescent fibroblasts were seeded in 96-well plates (353072; BD Biosciences, Franklin Lakes, NJ, USA) at a density of 1 × 10^3^ cells per well. Cells were then treated with DMSO (0.01%) or *P. cuspidatum* extract (1.25, 2.5, or 10 µg/mL) for 12 days. Cell numbers were enumerated using a cell proliferation assay based on a DNA content-based method [19,20,21]. Specifically, on day 12, cells were washed twice with phosphate-buffered saline (AM9624; Invitrogen, Waltham, MA, USA). A total of 50 µL of 0.2% sodium lauryl sulfate (L3771; Sigma) was added to each well and incubated at 37 °C for 1 h. Next, 150 μL of SYBR Green I nucleic acid gel stain (1:1000 in DW; Excitation/Emission: 485 nm/535 nm; S-7567; Molecular Probes, Eugene, OR, USA) was added to each well. Fluorescence intensity was measured using a VICTOR Multilabel Plate Reader (2030-0050; PerkinElmer, Waltham, MA, USA).

### 2.5. Analysis of the Oxygen Consumption Rate (OCR)

Senescent fibroblasts were treated with DMSO (0.01%) or *P. cuspidatum* extract (2.5 µg/mL) for 12 days. Analysis of the OCR was performed using the XFe24 flux analyzer (Seahorse Bioscience, Billerica, MA, USA). The analysis of OCR and the ATP production rate was performed as previously explained [20].

### 2.6. Flow Cytometric Analysis of Mitochondrial Membrane Potential (MMP), Cellular Lipofuscin Levels, Lysosomal Mass, and Autophagosome Level

Senescent fibroblasts were treated with DMSO (0.01%) or *P. cuspidatum* extract (2.5 µg/mL) for 12 days. To assess MMP, senescent fibroblasts were treated for 30 min at 37 °C in a medium containing 0.6 µg/mL JC-10 (ENZ-52305; Enzo Life Sciences, Farmingdale, NY, USA). One commonly used method for quantifying lipofuscin is autofluorescence assessment [22,23,24,25]. To assess autofluorescence, senescent fibroblasts were treated for 30 min at 37 °C in a medium containing no dyes. To assess lysosomal mass, senescent fibroblasts were treated for 30 min at 37 °C in a medium containing 100 nM LysoTracker™ Red (L7528; Thermo Fisher Scientific). To assess autophagosome level, senescent fibroblasts were treated for 30 min at 37 °C in a medium containing CYTO-ID^®^ (ENZ-51031-0050; Enzo Life Sciences). Then, cells were processed for flow cytometry analysis as previously explained [19].

### 2.7. Measurement of Protein Carbonylation

HaCaT cells were treated with 500 μM H_2_O_2_ (216763; Sigma) for 4 h. Then, HaCaT cells were treated with DMSO (0.01%) or *P. cuspidatum* extract (2.5 µg/mL) for 12 days. As a positive control, 250 µM vitamin E (T1539; Sigma) was used. Vitamin E was diluted to a concentration of 2.5 M using DMSO (D8418; Sigma). To make a concentration of 250 µM vitamin E, 1 μL of 2.5 M vitamin E was added to 10 mL medium. Then, HaCaT cells were treated with methanol for 5 min. HaCaT cells were stained with 20 μM fluorescein-5-thiosemicarbazide (46985; Sigma) and 4 μM hoechst 33342 (H21492; Thermo fisher scientific). Fluorescence was measured using an ELISA reader (Tecan, Männedorf, Switzerland). Cell morphology was photographed using a fluorescence microscope (Leica, Wetzlar, Germany).

### 2.8. Measurement of Calpain 1 Protein Expression after Activation with IL-17A

To inhibit the expression of calpain 1, HEKn cells were treated with 200 ng/mL IL-17A (200-17; Peprotech; Cranbury, NJ, USA). IL-17A was diluted to a concentration of 2 mg/mL using DMSO (D8418; Sigma). To make a concentration of 200 ng/mL IL-17A, 1 μL of 2 mg/mL IL-17A was added to 10 mL medium. Then, HEKn cells were treated with DMSO (0.01%) or *P. cuspidatum* extract (2.5 µg/mL) for 12 days. As a positive control, 500 μg/mL ceramide NP (EVONIK, Essen, Germany) was used. Ceramide NP was diluted to a concentration of 5 g/mL using DMSO (D8418; Sigma). To make a concentration of 500 μg/mL ceramide NP, 1 μL of 5 g/mL ceramide NP was added to 10 mL medium.

### 2.9. Measurement of Collagen Type I and III Expression

Senescent fibroblasts were treated with DMSO (0.01%) or *P. cuspidatum* extract (2.5 µg/mL) for 12 days. As a positive control, 75 µg/mL vitamin C (A4403; Sigma) was used. Vitamin C was diluted to a concentration of 750 mg/mL using DMSO (D8418; Sigma). To make a concentration of 75 µg/mL vitamin C, 1 μL of 750 mg/mL vitamin C was added to 10 mL medium.

### 2.10. Measurement of NLRP3 Protein Expression after Activation with LPS/ATP

To induce NLR family pyrin domain-containing 3 (NLRP3) expression, HaCaT cells were treated with 5 μg/mL lipopolysaccharide (LPS; L4516; Sigma) and then with 5 mM adenosine triphosphate (ATP; A6419; Sigma). LPS was diluted to a concentration of 50 mg/mL using DMSO (D8418; Sigma). To make the concentration of 5 μg/mL LPS, 1 μL of 50 mg/mL LPS was added to 10 mL medium. ATP was diluted to a concentration of 50 M using distilled water (07-6061; Sigma). To make the concentration of 5 mM ATP, 1 μL of 50 M ATP was added to 10 mL medium. Then, HaCaT cells were treated with DMSO (0.01%) or *P. cuspidatum* extract (2.5 µg/mL) for 12 days. As a ROS scavenger, 5 mM N-acetylcysteine (NAC; A9165; Sigma) was treated on HaCaT cells. NAC was diluted to a concentration of 50 M using DMSO (D8418; Sigma). To make the concentration of 5 mM NAC, 1 μL of 50 M ATP was added to 10 mL medium.

### 2.11. Measurement of IL-8 Expression after Activation with IFN-γ/TNF-α

To induce IL-8 expression, HaCaT cells were treated with 10 ng/mL interferon gamma (IFN-γ; PHC4031; Gibco) and 20 ng/mL tumor necrosis factor-alpha (TNF-α; H8916; Sigma). IFN-γ was diluted to a concentration of 100 μg/mL using DMSO (D8418; Sigma). To make the concentration of 10 ng/mL IFN-γ, 1 μL of 100 μg/mL IFN-γ was added to 10 mL medium. TNF-α was diluted to a concentration of 200 μM using distilled water (07-6061; Sigma). To make the concentration of 20 ng/mL TNF-α, 1 μL of 200 μM TNF-α was added to 10 mL medium. Then, HaCaT cells were treated with DMSO (0.01%) or *P. cuspidatum* extract (2.5 µg/mL) for 12 days.

### 2.12. Measurement of β-Defensin 2 Expression after Activation with IFN-γ/TNF-α Followed by IL-4

HaCaT cells were treated with 10 ng/mL IFN-γ and 20 ng/mL TNF-α. To inhibit the expression of β-defensin 2, HaCaT cells were treated with 50 ng/mL IL-4 (PHC0044; Gibco). IL-4 was diluted to a concentration of 500 μg/mL using DMSO (D8418; Sigma). To make the concentration of 50 ng/mL IL-4, 1 μL of 500 μg/mL IL-4 was added to 10 mL medium. Then, HaCaT cells were treated with DMSO (0.01%) or *P. cuspidatum* extract (2.5 µg/mL) for 12 days. As a positive control, 25 μg/mL lactoferrin (L4894; Sigma) was used. Lactoferrin was diluted to a concentration of 250 mg/mL using DMSO (D8418; Sigma). To make the concentration of 25 μg/mL lactoferrin, 1 μL of 250 mg/mL lactoferrin was added to 10 mL medium.

### 2.13. Quantitative PCR (qPCR)

qPCR using mRNA was performed as described previously [26]. qPCR was conducted using the following primer (Table 1).

### 2.14. Western Blot Analysis

The protocol for Western blot analysis was followed as previously described [27]. Antibodies used in this study included calpain 1 antibody (ab108400; Abcam, Cambridge, UK, 1:1000 dilution in 5% skim milk), GAPDH antibody (sc-32233; Santa Cruz Biotechnology, Dallas, TX, USA, 1:5000 dilution in 5% skim milk), NLRP3 antibody (PA5-79740; Invitrogen, 1:1000 dilution in 5% skim milk), β-actin antibody (sc-47778; Santa cruz biotechnology, 1:5000 dilution in 5% skim milk), Horseradish peroxidase (HRP)-conjugated secondary antibody (1706515; Bio-Rad, Hercules, CA, USA, 1:2000 dilution in 5% skim milk), and HRP-conjugated secondary antibody (1706516; Bio-Rad, 1:10,000 dilution in 5% skim milk).

### 2.15. High-Performance Liquid Chromatography (HPLC) Analysis

Analysis of emodin and polydatin content in the *P. cuspidatum* extract was conducted using HPLC (Agilent 1200, Agilent Technologies, Santa Clara, CA, USA). HPLC column (Capcell Pak C18 4.6 × 250 mm, Shiseido, Osaka, Japan) was used. A total of 10.1 mg of *P. cuspidatum* extract was diluted to a 25 mL methyl alcohol (1424109; Sigma). To make a standard solution, 4.6 mg of emodin (E7881; Sigma) or 7.6 mg of polydatin (15721; Sigma) was dissolved in 50 mL of methyl alcohol.

## 3. Results

### 3.1. P. cuspidatum Extract Significantly Reduces ROS Levels in Senescent Fibroblasts

**Figure 1:**
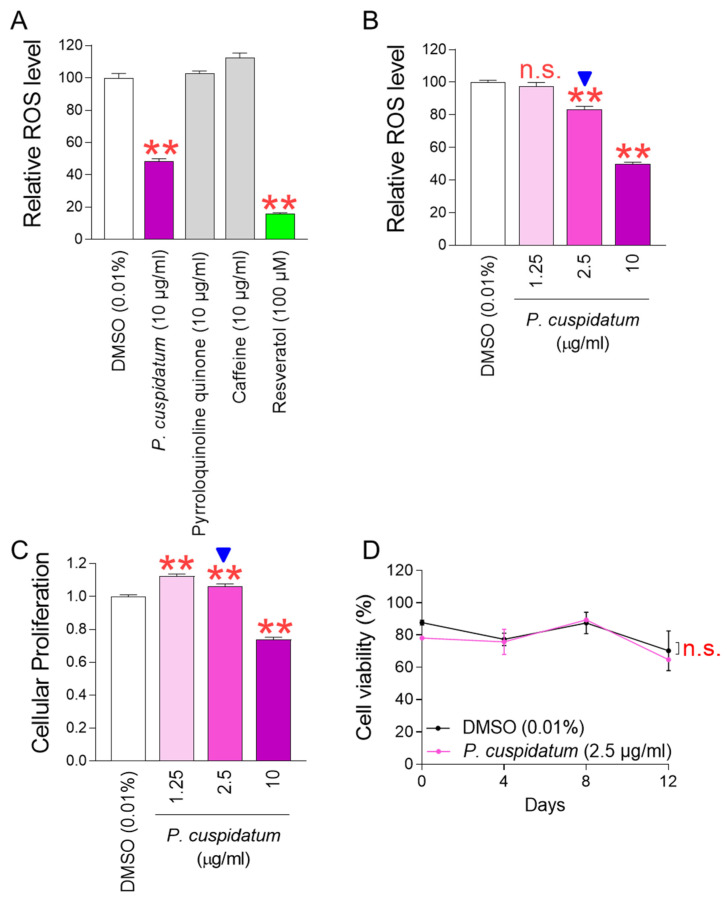
*P. cuspidatum* extract significantly reduces ROS levels in senescent fibroblasts. (**A**) Senescent fibroblasts were treated with *P. cuspidatum* extract (10 μg/mL), pyrroloquinoline quinone (PQQ) (10 μg/mL), and caffeine (10 μg/mL). On day 12, their impact on ROS levels was examined. DMSO control was used by diluting DMSO in the medium to a concentration of 0.01%. Flow cytometric analysis of ROS using DHR123. ** *p* < 0.01, Student’s *t*-test. Mean ± S.D., N = 3. Resveratrol (100 μM) was used as a positive control. (**B**) ROS levels were assessed at different concentrations of *P. cuspidatum* extract (1.25, 2.5, and 10 µg/mL) on day 12 after treatment in senescent fibroblasts. DMSO control was used by diluting DMSO in the medium to a concentration of 0.01%. n.s. (not significant), ** *p* < 0.01, Student’s *t*-test. Mean ± S.D., N = 3. (**C**) Cellular proliferation was assessed at different concentrations of *P. cuspidatum* extract (1.25, 2.5, and 10 µg/mL) on day 12 after treatment in senescent fibroblasts. DMSO control was used by diluting DMSO in the medium to a concentration of 0.01%. ** *p* < 0.01, Student’s *t*-test. Mean ± S.D., N = 3. The arrow head indicates the optimal concentration of *P. cuspidatum* extract on decreasing ROS levels and increasing cellular proliferation. (**D**) Measurement of cell viability after 0, 4, 8, and 12 days of treatment with DMSO (0.01%) or *P. cuspidatum* extract (2.5 µg/mL) in senescent fibroblasts. n.s. (not significant), two-way ANOVA followed by Bonferroni’s post hoc test. Mean ± S.D., N = 3.

Among the widely used cosmetic ingredients, *P. cuspidatum* extract, pyrroloquinoline quinone (PQQ), and caffeine were used to find candidates that could effectively suppress ROS in senescent fibroblasts. *P. cuspidatum* extract is known to be effective in skin soothing and whitening [17,18]. PQQ prevents skin aging by regenerating and thickening skin cells [28]. Caffeine protects the skin from ultraviolet rays, slowing down photoaging of the skin [29]. Three substances were diluted to a final concentration of 10 µg/mL and treated to senescent fibroblasts. Then, on day 12, their impact on ROS levels was examined. Resveratrol, known as a potent antioxidant, was used as a positive control. As expected, resveratrol significantly decreased ROS levels in senescent fibroblasts (Figure 1A). *P. cuspidatum* extract also significantly decreased ROS levels, exhibiting a ROS-reducing effect similar to resveratrol (Figure 1A). However, PQQ and caffeine, which are known to be effective in skin beauty, were not effective in reducing ROS levels (Figure 1A). These results suggest that among the three ingredients known to be effective in skin beauty, only the *P. cuspidatum* extract exhibited antioxidant activity (Figure 1A).

To determine the concentration at which *P. cuspidatum* extract simultaneously reduces ROS and senescence-associated phenotypes, senescent fibroblasts were treated with *P. cuspidatum* extract at concentrations of 0–10 μg/mL. Since the definition of senescence is the irreversible arrest of the cell cycle [30], the cell proliferation-inducing effect of *P. cuspidatum* extract was used as a criterion for the recovery of senescence-associated phenotypes. A significant ROS-reducing effect was observed starting from a concentration of 2.5 μg/mL, including the 10 μg/mL concentration used in the initial screening (Figure 1B). However, the cell proliferation-inducing effect was observed only at concentrations of 1.25 and 2.5 μg/mL (Figure 1C). Here, 2.5 μg/mL was chosen as the optimal concentration of *P. cuspidatum* extract because it simultaneously reduces ROS and increases cell proliferation in senescent fibroblasts (Figure 1B,C; blue arrow heads).

Next, we investigated the toxicity of *P. cuspidatum* at selected concentrations by examining cell viability. Senescent fibroblasts treated with *P. cuspidatum* extract at the concentration exhibited similar viability as fibroblasts treated with DMSO, suggesting that *P. cuspidatum* at selected concentrations was not toxic to the cells (Figure 1D).

### 3.2. P. cuspidatum Extract Ameliorates Senescence-Associated Phenotypes in Senescent Fibroblasts

Senescent fibroblasts treated with selected concentrations of *P. cuspidatum* extract showed proliferation-inducing effects, ameliorating irreversible cell cycle arrest, one of the senescence-associated phenotypes. However, since the recovery of senescence-associated phenotypes by *P. cuspidatum* extract should not be based solely on its cell proliferation-inducing effect, the effects of *P. cuspidatum* extract on the other senescence-associated phenotypes were also investigated.

**Figure 2:**
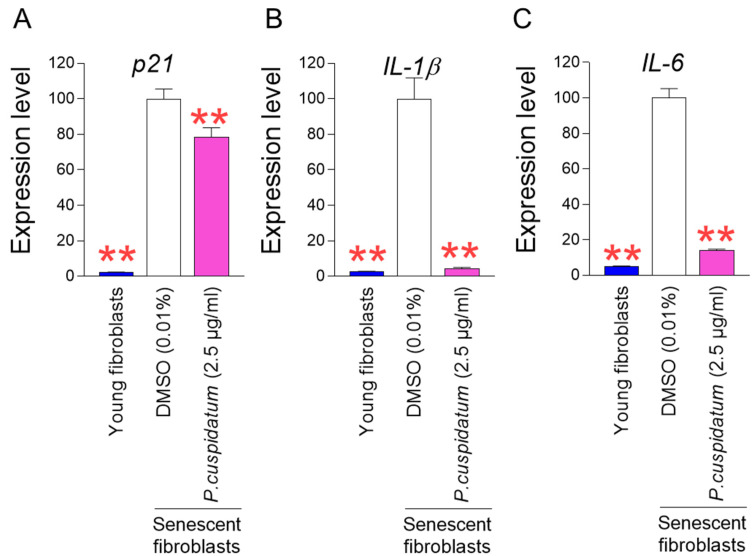
*P. cuspidatum* extract ameliorate senescence-associated phenotypes in senescent fibroblasts. (**A**) Expression levels of *p21* gene after 12 days of treatment with DMSO (0.01%) or *P. cuspidatum* extract (2.5 µg/mL) in senescent fibroblasts. ** *p* < 0.01, Student’s *t*-test. Mean ± S.D., N = 3. (**B**) Expression levels of *IL-1β* gene after 12 days of treatment with DMSO (0.01%) or *P. cuspidatum* extract (2.5 µg/mL) in senescent fibroblasts. ** *p* < 0.01, Student’s *t*-test. Mean ± S.D., N = 3. (**C**) Expression levels of *IL-6* after 12 days of treatment with DMSO (0.01%) or *P. cuspidatum* extract (2.5 µg/mL) in senescent fibroblasts. ** *p* < 0.01, Student’s *t*-test. Mean ± S.D., N = 3.

Two major pathways controlling cell cycle arrest, one of the senescence-associated phenotypes, are the p53/p21 and p16/RB pathways [31]. The p53/p21 pathway plays a key role in the early stages of senescence, whereas the p16/RB pathway plays a critical role in the maintenance of senescence [32]. Among the two major pathways, we selected the p53/p21 pathway and examined the changes in the expression of *p21*, a downstream pathway of *p53*. Young fibroblasts were used as a positive control, and *p21* expression in young fibroblasts was significantly lower than in senescent fibroblasts, as previously reported [20] (Figure 2A). *P. cuspidatum* treatment in senescent fibroblasts significantly reduced *p21* expression compared to DMSO control, indicating cell cycle progression by *P. cuspidatum* (Figure 2A).

The senescence-associated secretory phenotype (SASP) consists of cytokines and chemokines secreted by senescent cells [31]. ROS influences the secretion of inflammatory SASP [33]. Particularly, O_2_^•−^ reacts with mitochondrial superoxide dismutase in the matrix to generate hydrogen peroxide, which is able to pass through the mitochondrial outer membrane and oxidize proteins in the cytosol. This reaction promotes the release of SASPs (IL-1β, IL-6, IL-8) [34,35,36]. Among the SASPs, we first selected *IL-1β*, known as an inflammatory SASP that induces T helper immune responses [37], and examined the changes in *IL-1β* expression. *IL-1β* expression in young fibroblasts was significantly lower than in senescent fibroblasts, as reported in previous studies [38] (Figure 2B). *P. cuspidatum* treatment in senescent fibroblasts significantly decreased *IL-1β* expression compared to the DMSO control, indicating that *P. cuspidatum* extract reduces the expression of inflammatory SASPs (Figure 2B).

IL-6 is known as an inflammatory SASP that promotes senescence through the autocrine and paracrine pathways [39]. We then examined the changes in *IL-6* expression. As previously reported [40], *IL-6* expression in young fibroblasts was significantly lower than in senescent fibroblasts (Figure 2C). *P. cuspidatum* treatment in senescent fibroblasts significantly decreased *IL-6* expression compared to the DMSO control, supporting the role of *P. cuspidatum* extract in reducing inflammatory SASPs (Figure 2C).

### 3.3. P. cuspidatum Extract Reduces Mitochondrial ROS Generation through Increasing OXPHOS Efficiency in Senescent Fibroblasts

**Figure 3:**
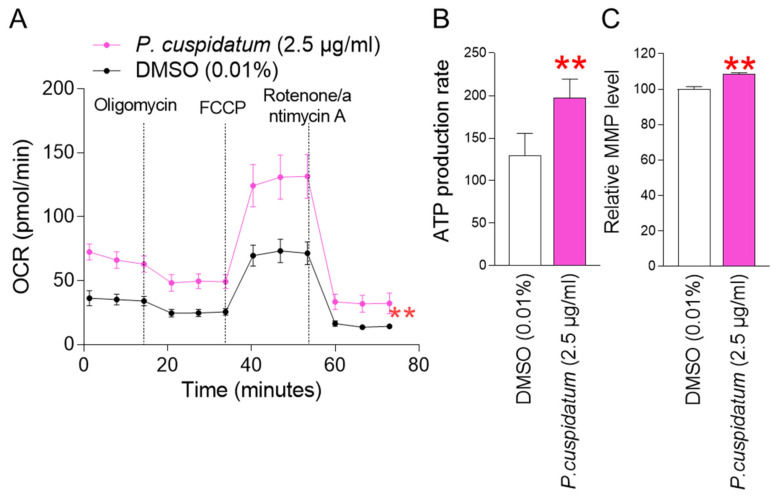
*P. cuspidatum* extract reduces mitochondrial ROS generation by increasing OXPHOS efficiency in senescent fibroblasts. (**A**) Measurement of oxygen consumption rate (OCR; pmole/min) after 12 days of treatment with DMSO (0.01%) or *P. cuspidatum* extract (2.5 µg/mL). (black line: DMSO-treated senescent fibroblasts, pink line: *P. cuspidatum*-extract-treated senescent fibroblasts). ** *p* < 0.01, two-way ANOVA followed by Bonferroni’s post hoc test. Means ± S.D., N = 3. (**B**) Measurement of ATP production rate after 12 days of treatment with DMSO (0.01%) or *P. cuspidatum* extract (2.5 µg/mL). ** *p* < 0.01, student *t*-test. Mean ± S.D., N = 3. (**C**) Measurement of mitochondrial membrane potential (MMP) after 12 days of treatment with DMSO (0.01%) or *P. cuspidatum* extract (2.5 µg/mL). ** *p* < 0.01, Student’s *t*-test. Means ± S.D., N = 3.

Inefficient electron transport in the mitochondrial electron transport chain (ETC) is a major cause of ROS production. In particular, inefficient electron transfer causes complexes I and III to generate superoxide anions (O_2_^•−^) from oxygen in the mitochondrial matrix [41]. It also causes complex III to generate O_2_^•−^ in the mitochondrial intermembrane space [41]. Since the mitochondrial ETC not only transfers electrons but also pumps protons from the matrix to the mitochondrial intermembrane space to enable oxidative phosphorylation (OXPHOS) [42], the electron transfer efficiency in the mitochondrial ETC can be indirectly measured through OXPHOS efficiency [43]. To understand the underlying mechanism of ROS reduction by *P. cuspidatum* extract, OXPHOS efficiency was investigated. Oxygen consumption rate (OCR; pmoles/min) was investigated as an indicator of OXPHOS efficiency [44]. Compound injections consisted of oligomycin, carbonyl cyanide-p-trifluoromethoxyphenylhydrazone (FCCP), and a combination of rotenone/antimycin A. ATP production (after oligomycin injection), maximal respiration (after FCCP injection), and non-mitochondrial respiration (after rotenone/antimycin A injection) were evaluated using the sequentially measured OCR values. Senescent fibroblasts treated with the *P. cuspidatum* extract showed significantly higher OCR values than the DMSO control after each injection, indicating that the *P. cuspidatum* extract enhanced OXPHOS efficiency (Figure 3A; black vs. pink lines).

Since we observed an increase in OXPHOS efficiency as a result of *P. cuspidatum* extract, we investigated whether *P. cuspidatum* extract could induce an increase in mitochondrial ATP production. *P. cuspidatum* extract increased ATP production compared to the DMSO control (Figure 3B). These results suggest that *P. cuspidatum* extract increased ATP production by improving the efficiency of OXPHOS.

Mitochondrial membrane potential (MMP) is the electrical potential that is generated when protons move from the matrix to the mitochondrial intermembrane space [45]. MMP is the electrical potential difference that drives ATP production in mitochondria [46]. Since we observed an increase in mitochondrial ATP production as a result of *P. cuspidatum* extract, we investigated the changes in MMP. *P. cuspidatum* extract increased MMP compared to the control (Figure 3C).

Considering the increase in OXPHOS efficiency and the resulting increase in ATP production as a result of *P. cuspidatum* extract, efficient electron transport induced by *P. cuspidatum* extract might be the underlying mechanism of reduction in mitochondrial ROS generation.

### 3.4. P. cuspidatum Extract Yields Functional Recovery of Lysosome/Autophagy System in Senescent Fibroblasts

**Figure 4:**
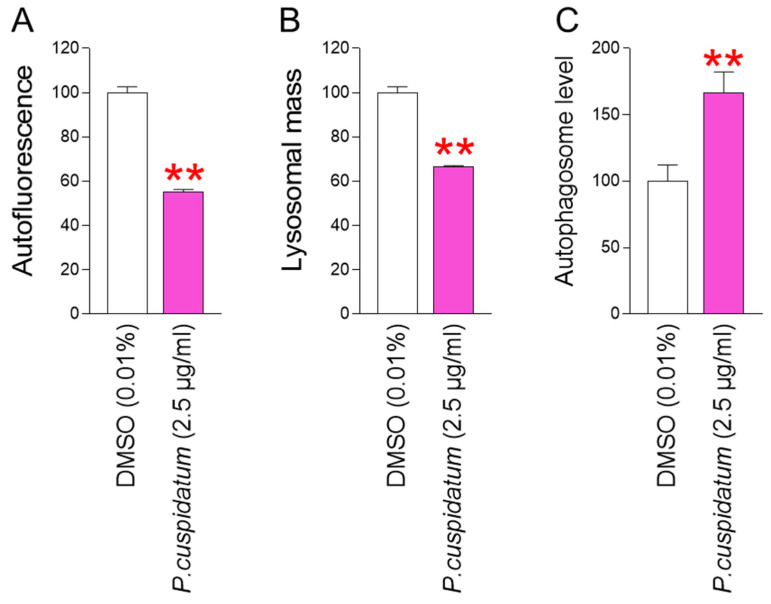
*P. cuspidatum* extract yields functional recovery of lysosome/autophagy system in senescent fibroblasts. (**A**) Autofluorescence was examined using flow cytometry after 12 days of treatment with DMSO (0.01%) or *P. cuspidatum* extract (2.5 µg/mL) in senescent fibroblasts. ** *p* < 0.01, Student’s *t*-test. Mean ± S.D., N = 3. (**B**) Measurement of lysosomal mass after 12 days of treatment with DMSO (0.01%) or *P. cuspidatum* extract (2.5 µg/mL) in senescent fibroblasts. ** *p* < 0.01, Student’s *t*-test. Mean ± S.D., N = 3. (**C**) Measurement of autophagosome level after 12 days of treatment with DMSO (0.01%) or *P. cuspidatum* extract (2.5 µg/mL) in senescent fibroblasts. ** *p* < 0.01, Student’s *t*-test. Mean ± S.D., N = 3.

Restoration of mitochondrial function is a prerequisite for improving senescence [19,47,48,49,50]. Increase in mitochondrial ATP production by *P. cuspidatum* extract prompted us to investigate its effects on senescence-associated phenotypes. We investigated the amount of intracellular lipofuscin, one of the senescence-associated phenotypes [28]. Lipofuscin levels were determined by assessing the amount of intracellular autofluorescence [51]. The autofluorescence levels were significantly reduced after *P. cuspidatum* treatment, suggesting that *P. cuspidatum* decreased lipofuscin levels (Figure 4A).

Lipofuscin is one type of autofluorescent material that gradually accumulates in lysosomes. Lysosomes filled with lipofuscin act as a sink for newly synthesized hydrolytic enzymes, thereby reducing lysosomal activity. The decrease in lysosomal activity leads to an increase in lysosomal mass to compensate for the decreased activity [52]. Therefore, we investigated the changes in lysosomal mass to determine whether *P. cuspidatum* extract affects lysosomal activity. Lysosomal mass was significantly decreased by *P. cuspidatum*, suggesting a reduction in nonfunctional lysosomes by *P. cuspidatum* (Figure 4B).

The lysosome/autophagy system plays a key role in the removal of dysfunctional mitochondria [53]. Given the observed reduction in lysosomal mass, we investigated whether the *P. cuspidatum* extract restored the autophagy system. *P. cuspidatum* extract increased the number of autophagosomes, indicating an activated autophagy system mediated by *P. cuspidatum* (Figure 4C).

### 3.5. P. cuspidatum Extract Reduces ROS and Lipofuscin Levels in Young Fibroblasts

**Figure 5:**
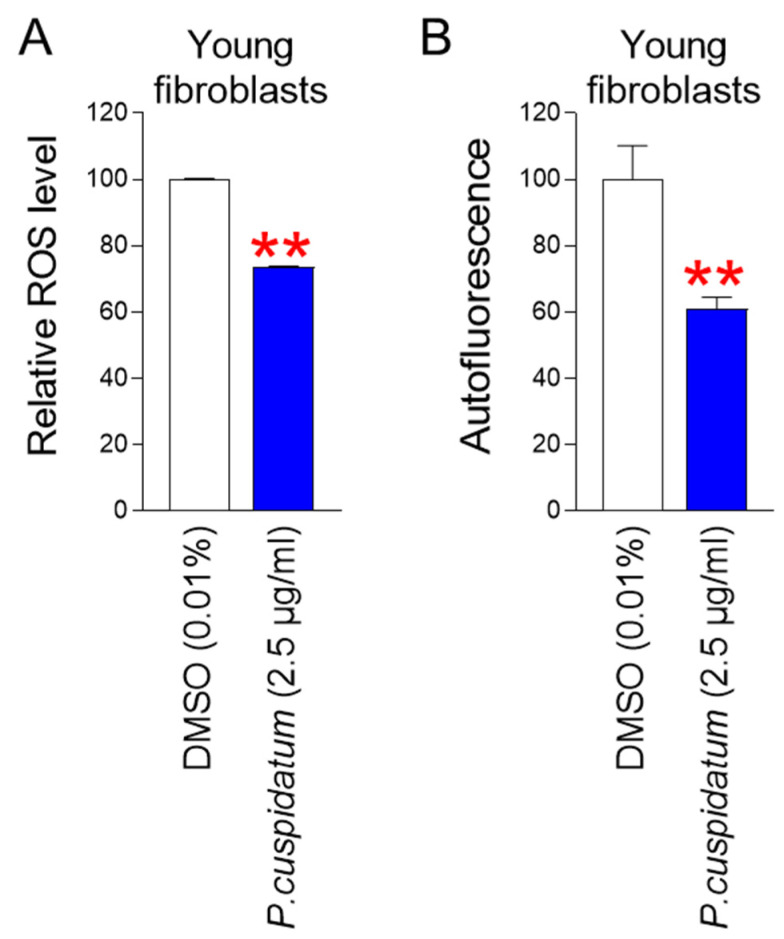
*P. cuspidatum* extract reduces ROS and lipofuscin levels in young fibroblasts. (**A**) ROS levels after 12 days of treatment with DMSO (0.01%) or *P. cuspidatum* extract (2.5 µg/mL) in young fibroblasts. ** *p* < 0.01, Student’s *t*-test. Mean ± S.D., N = 3. (**B**) Autofluorescence was examined using flow cytometry after 12 days of treatment with DMSO (0.01%) or *P. cuspidatum* extract (2.5 µg/mL) in young fibroblasts. ** *p* < 0.01, Student’s *t*-test. Mean ± S.D., N = 3.

Our results showing that *P. cuspidatum* extract improves senescence-associated phenotypes in senescent fibroblasts raise the question of whether these improvements also apply to young fibroblasts. Therefore, we investigated whether *P. cuspidatum* extract exhibited the same effect on young fibroblasts. First, we examined the effect of *P. cuspidatum* extract on ROS levels in young fibroblasts. *P. cuspidatum* extract exhibited ROS-reducing effects in young fibroblasts similar to those observed in senescent fibroblasts (Figure 5A). We then examined the effect of *P. cuspidatum* extract on intracellular lipofuscin levels in young fibroblasts. *P. cuspidatum* extract reduced autofluorescence levels in young fibroblasts, suggesting that *P. cuspidatum* also reduced lipofuscin levels in young cells, as was seen in senescent fibroblasts (Figure 5B). Taken together, these results suggest that the effects of *P. cuspidatum* are not limited to senescent fibroblasts.

### 3.6. P. cuspidatum Extract Enhances Skin Protection through Restoring Skin Barrier Formation

**Figure 6:**
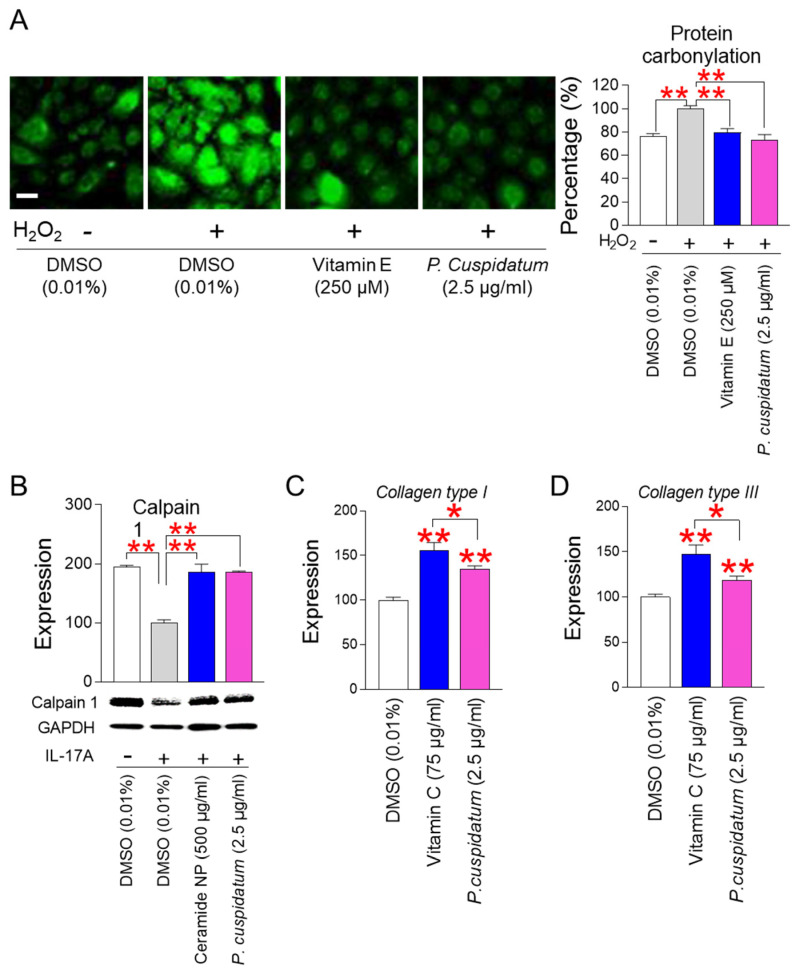
*P. cuspidatum* extract enhances skin protection by restoring skin barrier formation. (**A**) Measurement of protein carbonylation. Normal human epidermal keratinocytes, HEKn cells, were treated with 500 μM H_2_O_2_ for 4 h. Then, HEKn cells were treated with DMSO (0.01%) or *P. cuspidatum* extract (2.5 µg/mL) for 12 days. As a positive control, vitamin E (250 µM; T1539; Sigma) was used. ** *p* < 0.01, Student’s *t*-test. Mean ± S.D., N = 3. Scale bar: 10 μm. Full-size images of immunofluorescence are shown in Supplementary Information S1. (**B**) Expression levels of calpain 1 protein after activation with IL-17A. To inhibit the expression of calpain 1, HEKn cells were treated with 200 ng/mL IL-17A. Then, HEKn cells were treated with DMSO (0.01%) or *P. cuspidatum* extract (2.5 µg/mL) for 12 days. As a positive control, ceramide NP (500 μg/mL) was used. ** *p* < 0.01, Student’s *t*-test. Mean ± S.D., N = 3. Full-size images of western blot are shown in Supplementary Information S2. (**C**) Expression levels of *collagen type I* after 12 days of treatment with DMSO (0.01%) or *P. cuspidatum* extract (2.5 µg/mL). Senescent fibroblasts were treated with DMSO (0.01%) or *P. cuspidatum* extract (2.5 µg/mL) for 12 days. As a positive control, vitamin C (75 µg/mL) was used. * *p* < 0.05, ** *p* < 0.01, Student’s *t*-test. Mean ± S.D., N = 3. (**D**) Expression level of *collagen type III* after 12 days of treatment with DMSO (0.01%) or *P. cuspidatum* extract (2.5 µg/mL). Senescent fibroblasts were treated with DMSO (0.01%) or *P. cuspidatum* extract (2.5 µg/mL) for 12 days. As a positive control, vitamin C (75 µg/mL) was used. * *p* < 0.05, ** *p* < 0.01, Student’s *t*-test. Mean ± S.D., N = 3.

Carbonylated proteins generated by ROS-induced protein oxidation are known to be one of the main causes of skin barrier damage in the stratum corneum [54]. To investigate the effect of *P. cuspidatum* on protein carbonylation, normal human epidermal keratinocytes, HEKn cells, were used. HEKn cells were treated with hydrogen peroxide to induce protein carbonylation. As a positive control, vitamin E, commonly used as an antioxidant, was used [55]. Hydrogen peroxide treatment significantly increased protein carbonylation (Figure 6A, Supplementary Information S1). However, the antioxidant vitamin E significantly reduced protein carbonylation (Figure 6A, Supplementary Information S1). As seen for vitamin E, the level of protein carbonylation was significantly reduced by *P. cuspidatum*, indicating that *P. cuspidatum* reduced ROS-induced protein oxidation (Figure 6A, Supplementary Information S1).

Calpain 1 plays an important role in skin barrier formation through its role as an inhibitor of various inflammatory pathways [56,57]. IL-17A promotes skin inflammation by inducing various inflammatory cytokines and activating T cells [58,59]. Ceramide NP, which has a structure similar to the skin lipid barrier and is known to strengthen the lipid barrier of aged skin, was used as a positive control [60]. To investigate the role of *P. cuspidatum* extract on calpain 1 expression, HEKn cells were activated with IL-17A. IL-17A treatment significantly reduced the expression of calpain 1 protein, indicating IL-17A-mediated induction of skin inflammation (Figure 6B, Supplementary Information S2). Ceramide NP, used as a positive control, subtly increased calpain 1 expression (Figure 6B, Supplementary Information S2). However, the expression level of calpain 1 protein was significantly increased by *P. cuspidatum*, indicating that the *P. cuspidatum* extract restored skin barrier formation through its role as an inhibitor of skin inflammation (Figure 6B, Supplementary Information S2).

Collagen is a major component of dermal extracellular matrix. Changes in collagen content in the dermal are the driving force for skin aging [61]. Collagen types I and III are essential for skin tissue regeneration, and type III collagen synthesis increases in the early stage of skin regeneration, while type I collagen synthesis increases in the late stage of skin regeneration [62]. To investigate the role of *P. cuspidatum* extract on skin tissue regeneration, senescent fibroblasts were treated with *P. cuspidatum* extract. *P. cuspidatum* extract significantly increased the expression of *collagen types I and III*, indicating that *P. cuspidatum* is very effective in the early and late stages of skin regeneration (Figure 6C,D). As a positive control, we used vitamin C, which is an antioxidant and also participates in the redox recycling of other antioxidants [63]. Vitamin C, an antioxidant, significantly increased the expression of *collagen types I and III*, and the degree of increase was higher than that induced by *P. cuspidatum* extract (Figure 6C,D).

### 3.7. P. cuspidatum Extract Enhances Skin Protection through Inhibiting Skin Inflammation

**Figure 7:**
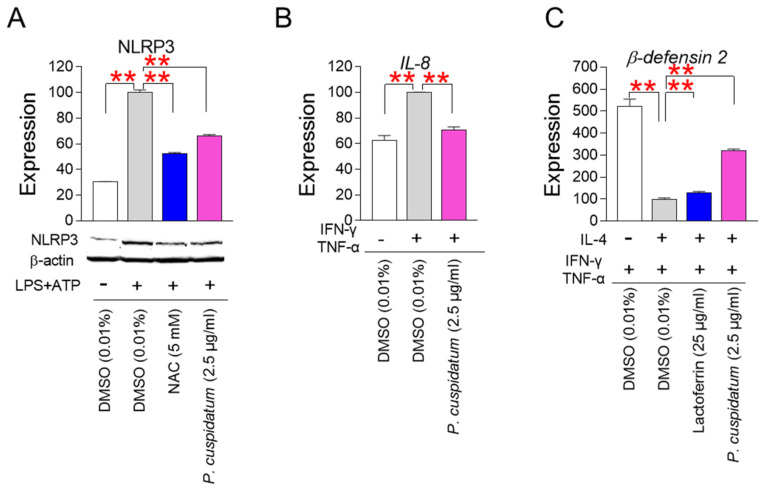
*P. cuspidatum* extract enhances skin protection by inhibiting skin inflammation. (**A**) Expression of NLRP3 protein after activation with lipopolysaccharide (LPS)/adenosine triphosphate (ATP). HaCaT cells were treated with 5 μg/mL LPS and then with 5 mM ATP. Then, HaCaT cells were treated with DMSO (0.01%) or *P. cuspidatum* extract (2.5 µg/mL) for 12 days. As a ROS scavenger, 5 mM N-acetylcysteine (NAC) was treated on HaCaT cells. ** *p* < 0.01, Student’s *t*-test. Mean ± S.D., N = 3. Full-size images of western blot are shown in Supplementary Information S2. (**B**) Expression levels of *IL-8* after activation with interferon gamma (IFN-γ)/tumor necrosis factor alpha (TNF-α). HaCaT cells were treated with 10 ng/mL IFN-γ and 20 ng/mL TNF-α. Then, HaCaT cells were treated with DMSO (0.01%) or *P. cuspidatum* extract (2.5 µg/mL) for 12 days. ** *p* < 0.01, Student’s *t*-test. Mean ± S.D., N = 3. (**C**) Expression levels of *β-defensin 2* after activation with IFN-γ/TNF-α followed by IL-4. HaCaT cells were treated with 10 ng/mL IFN-γ and 20 ng/mL TNF-α. Then, HaCaT cells were treated with 50 ng/mL IL-4. HaCaT cells were treated with DMSO (0.01%) or *P. cuspidatum* extract (2.5 µg/mL) for 12 days. As a positive control, 25 μg/mL lactoferrin was used. ** *p* < 0.01, Student’s *t*-test. Mean ± S.D., N = 3.

NLR family pyrin domain-containing 3 (NLRP3) plays a key role in regulating the innate immune system and inflammatory signaling [64]. NLRP3 is activated by lipopolysaccharide (LPS)/adenosine triphosphate (ATP) [65]. Since NLRP3 activation acts as an inducer of human autoimmune skin diseases, we investigated the effect of *P. cuspidatum* extract on NLRP3 levels. N-acetylcysteine (NAC), a ROS scavenger known to downregulate the NLRP3 inflammasome complex, was used as a positive control [66]. Activation of HaCaT cells by LPS/ATP significantly increased the expression of the NLRP3 protein (Figure 7A, Supplementary Information S2). However, NAC treatment significantly decreased NLRP3 expression (Figure 7A, Supplementary Information S2). Furthermore, the expression level of NLRP3 was significantly decreased by *P. cuspidatum*, indicating that the *P. cuspidatum* extract inhibited skin inflammation by downregulating NLRP3 expression (Figure 7A, Supplementary Information S2).

IL-8 is an inflammatory factor that plays a crucial role in skin inflammation [67]. IFN-γ and TNF-α, two cytokins released by activated T cells, synergistically activate IL-8 expression [68]. Activation of HaCaT cells by IFN-γ/TNF-α significantly upregulated *IL-8* expression (Figure 7B). However, *P. cuspidatum* extract significantly reduced *IL-8* expression levels, indicating that *P. cuspidatum* is very effective in preventing skin inflammation (Figure 7B).

β-defensin 2 is an antimicrobial peptide found in lesional skin and exhibits potent antimicrobial activity [69]. β-defensin 2 expression is increased by IFN-γ/TNF-α-mediated inflammation [70]. By contrast, IL-4 negatively regulates IFN-γ/TNF-α-induced β-defensin expression through activating signal transducer and activator of transcription 6 [71]. As a positive control, we used lactoferrin, which exhibits antimicrobial activity by limiting the amount of ions available for microbial metabolism [72]. To investigate the role of *P. cuspidatum* extract in *β-defensin 2* expression, HaCaT cells activated by IFN-γ/TNF-α were treated with IL-4. IL-4 treatment decreased *β-defensin 2* expression induced by IFN-γ/TNF-α (Figure 7C). However, lactoferrin exhibiting antimicrobial activity significantly increased *β-defensin 2* expression (Figure 7C). As seen for lactoferrin, the expression level of *β-defensin 2* was significantly increased by *P. cuspidatum*, indicating that the *P. cuspidatum* extract enhanced skin protective function by increasing antibacterial activity (Figure 7C).

### 3.8. Identification of Emodin and Polydatin from P. cuspidatum Extracts

**Figure 8:**
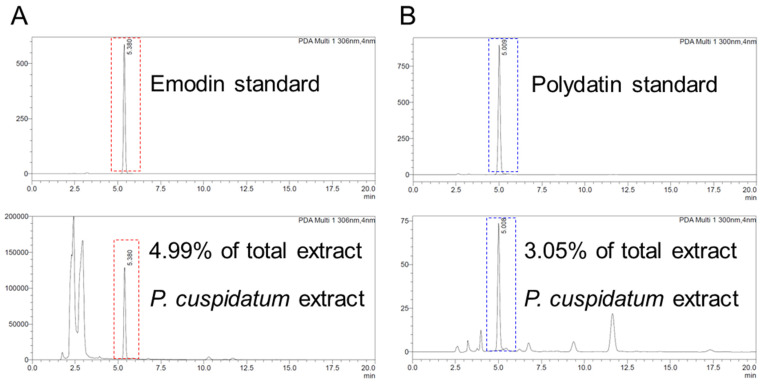
Identification of emodin and polydatin from *P. cuspidatum* extracts. High-performance liquid chromatography (HPLC) was performed to determine how much polydatin and emodin were present in *P. cuspidatum* extract. (**A**) The HPLC peak of the *P. cuspidatum* extract matched the emodin standard, and the amount of emodin present in the *P. cuspidatum* extract was 4.99%. (**B**) The HPLC peak of the *P. cuspidatum* extract matched the polydatin standard, and its amount was 3.05% of the total extract.

The discovery that *P. cuspidatum* extract is effective in enhancing skin protection by reducing oxidative stress led us to identify active ingredients with antioxidant effects among the components present in *P. cuspidatum* extract. Phytochemical components of *P. cuspidatum* extract were known to be phenolic compounds, stilbene derivatives, anthraquinone derivatives, and flavonoid compounds [73]. A literature review was conducted focusing on the antioxidant effects of phytochemical components. Two ingredients, emodin and polydatin, have been known to have antioxidant effects. Emodin, an anthraquinone derivative, has powerful antioxidant effects that may be useful against cancer [74]. Polydatin contains stilbenoid polyphenols, which act as singlet oxygen scavengers through redox reactions [75]. Therefore, first, liquid chromatography was performed to determine how much polydatin and emodin were present in the *P. cuspidatum* extract. The HPLC peak of the *P. cuspidatum* extract matched the emodin standard, and the amount of emodin present in the *P. cuspidatum* extract was 4.99% (Figure 8A). These results were comparable with previously tested and published results (3.37% ± 0.15%, N = 26) [76]. The HPLC peak of the *P. cuspidatum* extract matched the polydatin standard, and its amount was 3.05% of the total extract (Figure 8B). These results were comparable with previously tested and published results (4.08%, N = 1) [14].

### 3.9. Identification of Polydatin as an Active Ingredient Showing Antioxidant Effects

**Figure 9:**
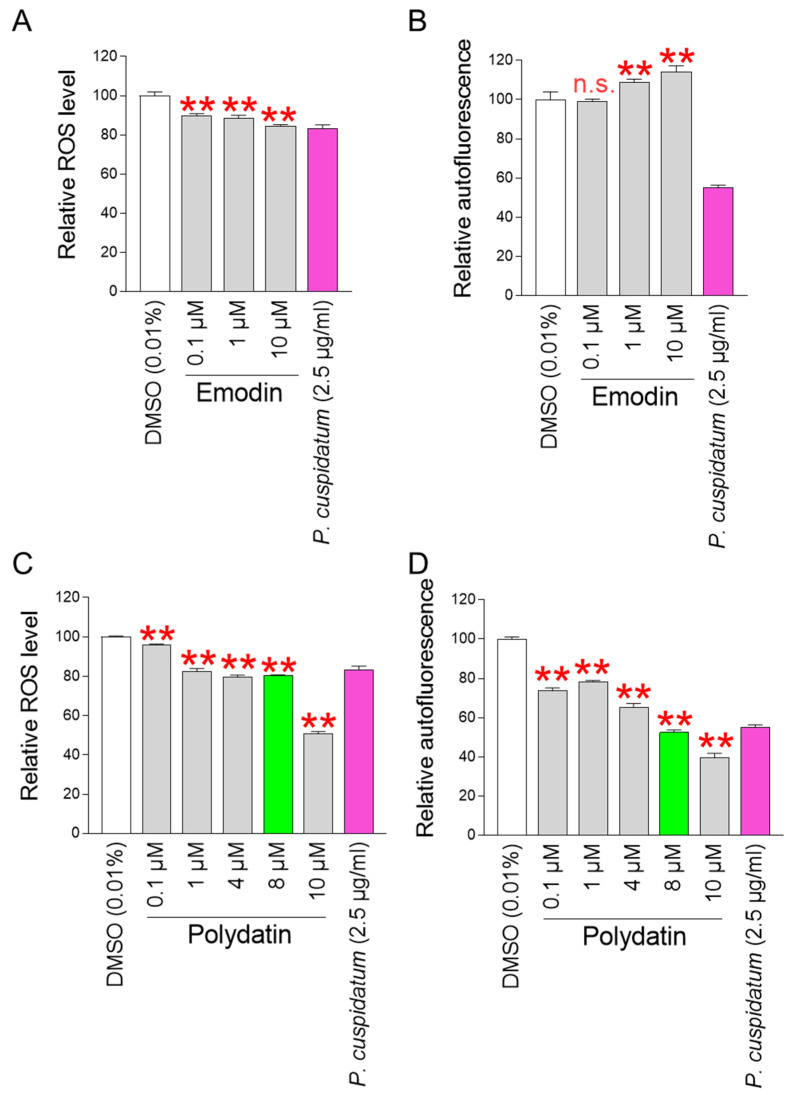
Identification of polydatin as an active ingredient showing antioxidant effects. (**A**) ROS-reducing effect of emodin was observed at concentrations of 0.1, 1, and 10 μM. As a positive control, senescent fibroblasts were treated with *P. cuspidatum* extract (2.5 µg/mL) for 12 days. ** *p* < 0.01, Student’s *t*-test. Mean ± S.D., N = 3. (**B**) The ROS-reducing effect of emodin were not sufficient to reduce the level of autofluorescence. As a positive control, senescent fibroblasts were treated with *P. cuspidatum* extract (2.5 µg/mL) for 12 days. n.s. (not significant), ** *p* < 0.01, Student’s *t*-test. Mean ± S.D., N = 3. (**C**) Polydatin was effective in reducing ROS levels at concentrations of 0.1, 1, 4, 8, and 10 μM. As a positive control, senescent fibroblasts were treated with *P. cuspidatum* extract (2.5 µg/mL) for 12 days. ** *p* < 0.01, Student’s *t*-test. Mean ± S.D., N = 3. (**D**) Polydatin was effective in reducing autofluorescence levels at concentrations of 0.1, 1, 4, 8, and 10 μM. As a positive control, senescent fibroblasts were treated with *P. cuspidatum* extract (2.5 µg/mL) for 12 days. A total of 8 μM polydatin was chosen as the optimal concentration because it is the minimum concentration more effective than *P. cuspidatum* extract. ** *p* < 0.01, Student’s *t*-test. Mean ± S.D., N = 3.

To identify active ingredients that exhibit antioxidant effects, senescent fibroblasts were treated with various concentrations of emodin and polydatin. Then, their effects on ROS levels and subsequent effects on senescence-associated phenotypes were evaluated. Autofluorescence, which is used to measure the amount of intracellular lipofuscin, a senescence-associated phenotype, was measured [51]. *P. cuspidatum* extract was used as a positive control. The significant ROS-reducing effect of emodin was observed at concentrations of 0.1, 1, and 10 μM (Figure 9A). However, the ROS-reducing effect of emodin did not reduce the level of autofluorescence, indicating that the antioxidant effects of emodin were not sufficient to reduce the senescence-associated phenotype (Figure 9B).

Polydatin was effective in reducing ROS levels at concentrations of 0.1, 1, 4, 8, and 10 μM (Figure 9C). Furthermore, polydatin was effective in reducing autofluorescence levels at concentrations of 0.1, 1, 4, 8, and 10 μM (Figure 9D). In particular, at 8 and 10 μM concentrations, polydatin was more effective in reducing ROS levels and autofluorescence levels than *P. cuspidatum* extract. Based on these data, polydatin was chosen to be an active ingredient that exhibits antioxidant effects and reduces the senescence-associated phenotype. 8 μM polydatin was selected as the optimal concentration for the following experiments because it was the minimal concentration that was more effective than *P. cuspidatum* extract.

### 3.10. Polydatin Reduces Mitochondrial ROS Generation by Increasing OXPHOS Efficiency

**Figure 10:**
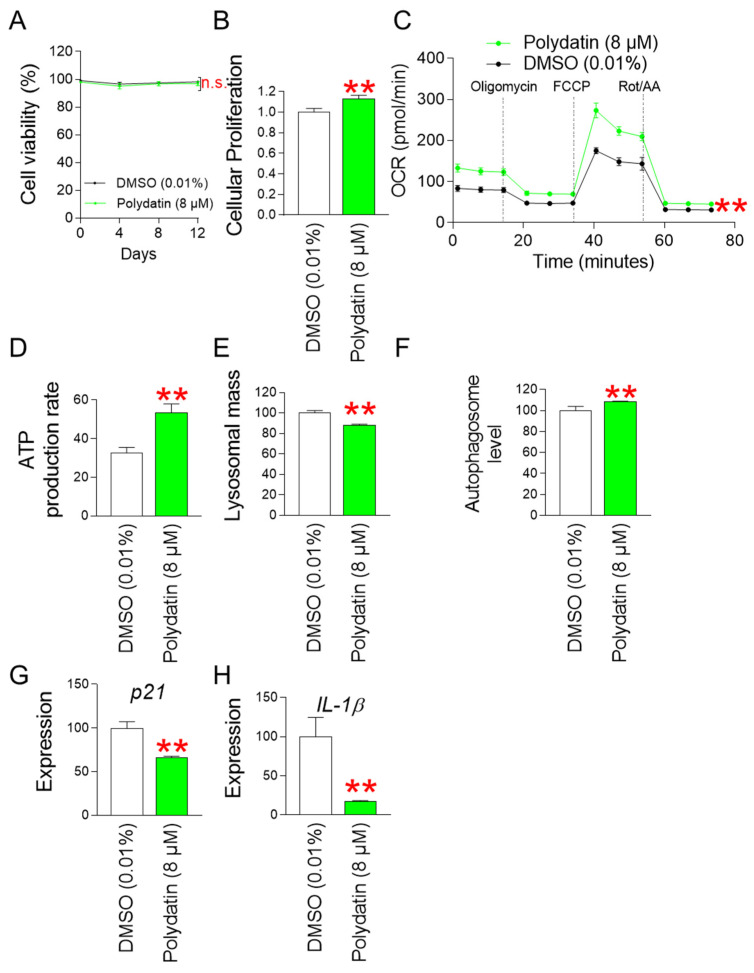
Polydatin reduces mitochondrial ROS generation through increasing OXPHOS efficiency. (**A**) Measurement of cell viability after 0, 4, 8, and 12 days of treatment with DMSO (0.01%) or polydatin (8 µM). n.s. (not significant), two-way ANOVA followed by Bonferroni’s post hoc test. Mean ± S.D., N = 3. (**B**) Measurement of cellular proliferation after 12 days of treatment with DMSO (0.01%) or polydatin (8 µM). ** *p* < 0.01, Student’s *t*-test. Mean ± S.D., N = 3. (**C**) Measurement of oxygen consumption rate (OCR; pmole/min) after 12 days of treatment with DMSO (0.01%) or polydatin (8 µM) (black line: DMSO-treated senescent fibroblasts, pink line: polydatin-treated senescent fibroblasts). ** *p* < 0.01, two-way ANOVA followed by Bonferroni’s post hoc test. Means ± S.D., N = 3. (**D**) Measurement of ATP production rate after 12 days of treatment with DMSO (0.01%) or polydatin (8 µM). ** *p* < 0.01, student *t*-test. Mean ± S.D., N = 3. (**E**) Measurement of lysosomal mass after 12 days of treatment with DMSO (0.01%) or polydatin (8 µM). ** *p* < 0.01, Student’s *t*-test. Mean ± S.D., N = 3. (**F**) Measurement of autophagosome level after 12 days of treatment with DMSO (0.01%) or polydatin (8 µM). ** *p* < 0.01, Student’s *t*-test. Mean ± S.D., N = 3. (**G**) Expression levels of *p21* after 12 days of treatment with DMSO (0.01%) or polydatin (8 µM). ** *p* < 0.01, Student’s *t*-test. Mean ± S.D., N = 3. (**H**) Expression levels of *IL-1β* after 12 days of treatment with DMSO (0.01%) or polydatin (8 µM). ** *p* < 0.01, Student’s *t*-test. Mean ± S.D., N = 3.

Next, we investigated the toxicity of polydatin at selected concentrations by examining cell viability. Senescent fibroblasts treated with polydatin exhibited similar viability as fibroblasts treated with DMSO, suggesting that polydatin at selected concentrations was not toxic to the cells (Figure 10A). We then examined the effect of polydatin on cellular proliferation at the selected concentration. Senescent fibroblasts treated with polydatin showed a cell proliferation-inducing effect similar to that induced by *P. cuspidatum* extract (Figure 10B).

Since we observed an increase in OXPHOS efficiency by *P. cuspidatum*, we also investigated the effect of polydatin on OXPHOS efficiency. Polydatin increased OCR levels, confirming the increased OXPHOS efficiency by polydatin (Figure 10C). As a result, polydatin-mediated increase in OXPHOS efficiency led to an increase in ATP production rate (Figure 10D).

Since we observed restoration of the lysosomal/autophagic system by *P. cuspidatum* at the autophagosome level, we investigated the effect of polydatin on the lysosomal/autophagic system. Polydatin decreased lysosomal mass levels and increased autophagosome levels (Figure 10E,F). These data imply that polydatin plays an important role in the restoration of the lysosomal/autophagic system.

The restoration of mitochondrial and lysosomal function by polydatin led us to investigate the effects of polydatin on other senescence-associated phenotypes. Polydatin significantly reduced *p21* expression compared to DMSO control, indicating cell cycle progression induced by polydatin (Figure 10G). Polydatin also significantly reduced *IL-1β* expression compared to DMSO control, suggesting that polydatin reduces anti-inflammatory cytokines (Figure 10H).

## 4. Discussion

Oxidative stress induced by ROS is closely linked to the onset and progression of aging [77]. ROS irreversibly damages proteins, lipids, DNA, and RNA, causing aging and age-related diseases [78]. A causal link between excessive oxidative stress and aging is supported by studies in the *Caenorhabditis elegans* aging model, which showed that increased oxidative stress by ROS causes physiological changes associated with premature aging, such as the accumulation of lipofuscin and carbonylated proteins [79,80]. Additional studies using mice deficient in superoxide dismutase 1 (SOD1), which is present in the mitochondrial intermembrane space and matrix, support this causal relationship [81]. SOD1 deficiency increased O_2_^•−^ formation and subsequent oxidative damage, leading to aging through a premature loss of skeletal muscle [81]. Mitochondria are the major organelles that generate oxidative stress within cells. More than 90% of oxygen is consumed in mitochondria, and 1–5% of oxygen is changed to highly reactive O_2_^•−^ by complexes I and III present in the ETC [41]. In the mitochondrial matrix, complexes I and III change oxygen into the O_2_^•−^. O_2_^•−^ is also generated by complex III in the intermembrane space of mitochondria. Mitochondrial dysfunction due to senescence reduces the activity of complexes present in the ETC, particularly complex I [82,83]. Complex I with impaired function cannot perform efficient electron transport, increases electron leakage to oxygen, and generates O_2_^•−^ [82,83]. Increased mitochondrial ROS production makes mitochondria targets for mitochondrial oxidative stress, further increasing mitochondrial ROS production [42]. A vicious loop between mitochondrial ROS generation and oxidative stress exacerbates the morphology and function of cellular organelles, ultimately causing senescence [84]. This causal relationship highlights the reduction in oxidative stress as an effective strategy to ameliorate senescence [42]. In this study, we discovered a novel mechanism by which *P. cuspidatum* reduced mitochondrial ROS production through increasing mitochondrial OXPHOS efficiency. Enhanced OXPHOS efficiency by *P. cuspidatum* extract suggests efficient electron transport in the mitochondrial ETC [85,86]. Since efficient electron transport promotes proton transport from the mitochondrial matrix to the mitochondrial intermembrane space, thereby increasing MMP [87], this is indirectly supported by the increased MMP by *P. cuspidatum* extract. Increased MMP increased mitochondrial ATP production [88,89], suggesting restoration of mitochondrial function by *P. cuspidatum* extract. Furthermore, the restoration of mitochondrial function by *P. cuspidatum* extract led to the recovery of several aging-related phenotypes [90,91]. Therefore, our results demonstrate for the first time that *P. cuspidatum* extract reverses senescence by reducing mitochondrial ROS production. We propose that reducing oxidative stress using *P. cuspidatum* extract might be a first step toward an effective therapeutic strategy against aging and age-related diseases.

Skin aging is one of the most obvious signs of aging [92]. Oxidative stress caused by free radicals is known to be the main cause of skin aging [93]. Therefore, antioxidants that balance unstable free radicals that damage skin tissues, especially collagen, have been widely used to delay or prevent skin aging [94]. For example, vitamin C, known as an antioxidant, has been shown to reduce free radical production and minimize damage to natural collagen in skin tissues [95]. In addition, niacinamide, an antioxidant, has been shown to protect skin from free radical damage, smooth uneven skin texture, and reduce the appearance of skin moisture [96]. In this study, we found that the reduction in mitochondrial ROS production by *P. cuspidatum* extract played an important role in the restoration of skin barrier formation. Specifically, *P. cuspidatum* extract reduced carbonylated proteins that damage the skin barrier in the stratum corneum [97]. The restoration of skin barrier formation by *P. cuspidatum* extract was evidenced by the increased expression of calpain 1 and major collagen types. Extending the relevance of these findings, this restoration was further supported by the enhancement of skin immune defense through the reduced expression of inflammatory factors and increased antimicrobial activity. Taken together, our findings suggest that reducing mitochondrial ROS production by *P. cuspidatum* extract might be an effective therapeutic strategy to enhance skin protection by restoring skin barrier formation and inhibiting skin inflammation.

Natural substances are widely used as cosmetic additives because they have a lower risk of causing adverse effects on the skin than synthetically manufactured compounds [98]. Various natural substances are added to cosmetics and are known to be effective in restoring skin aging [99]. However, since natural substances contain various ingredients, identifying effective ingredients is a very important step. Identifying effective ingredients can minimize the use of unnecessary or harmful substances contained in natural substances for restoring skin aging [100]. In addition, the ingredients of natural substances may change due to changes in the cultivation site or climate [101]. Such changes in the ingredients of natural substances can also cause changes in the performance of cosmetics that use natural substances as additives [99,102]. In this study, we confirmed that the antioxidant active component of *P. cuspidatum* extract was polydatin. Polydatin is a stilbenoid polyphenol and a resveratrol derivative with more biological activity [75]. Since the phenol group acts as a reducing agent, hydrogen donor, and singlet oxygen scavenger through redox reactions [103], the stilbenoid polyphenol of polydatin plays a leading role in the antioxidant effect [75]. In fact, polydatin was confirmed to significantly reduce ROS levels in senescent fibroblasts, similar to *P. cuspidatum* extract. This reduction may be due to the antioxidant effect of the stilbenoid polyphenol of polydatin, although we have not directly verified this. In addition, we found that this reduction was due to polydatin increasing OXPHOS efficiency and reducing mitochondrial ROS production, similar to the *P. cuspidatum* extract. To the best of our knowledge, this is the first study to show that polydatin reduces mitochondrial ROS production through increasing OXPHOS efficiency. We propose that the use of polydatin as a cosmetic additive can be effective in reversing skin aging while minimizing potential problems that may arise when using natural substances.

Pathological levels of ROS are known to permanently damage proteins, lipids, DNA, and RNA, which is ultimately one of the major causes of aging and age-related diseases [104]. Specifically, the accumulation of oxidative damage caused by ROS induces abnormalities in p53 signaling function, leading to prolonged cell cycle arrest and subsequent senescence [6,105,106,107,108]. In addition, excessive ROS induces collagen and elastin chain cleavage, which worsens skin aging [6]. Consequently, therapeutic strategies that reduce ROS levels are emerging as one of the effective treatments for anti-aging and age-related diseases [42]. However, physiological levels of ROS serve as physiological mediators and important second messenger signaling molecules in various cellular and developmental processes [109]. In particular, ROS within the physiological nanomolar concentration range induces the reversible oxidation of protein targets to regulate cellular metabolism and stress responses [110]. Moreover, physiological levels of ROS can induce adaptive responses that enhance endogenous antioxidant defenses, reduce long-term oxidative damage, and improve overall stress tolerance [111]. The importance of physiological levels of ROS is further supported by the finding that mitohormesis, a systemic adaptive response that promotes longevity, is regulated by moderate levels of ROS [112]. In this study, we revealed a novel mechanism by which *P. cuspidatum* extract reduces ROS by increasing OXPHOS efficiency. Next, we found that the active component of *P. cuspidatum* extract with antioxidant properties is polydatin. Effectively reducing ROS levels using *P. cuspidatum* extract and polydatin is an effective treatment to reverse senescence, but reducing ROS levels below physiological levels may have hazardous consequences and should be approached with caution.

*P. cuspidatum* extract has been confirmed to enhance skin protection by restoring skin barrier formation and suppressing skin inflammation. However, further studies are needed to directly utilize *P. cuspidatum* extract as a cosmetic raw material. First, although the efficacy was observed at 2.5 μg/mL, a concentration optimization process of *P. cuspidatum* extract is necessary for use in cosmetics. For example, the minimum concentration that shows skin whitening, skin regeneration, and skin elasticity effects without toxicity should be determined after serially diluting the substance and applying it to each test [113,114]. Then, the minimum concentration of *P. cuspidatum* extract should be applied when formulating cosmetics. Second, studies should be conducted to determine how well *P. cuspidatum* extract or its active ingredient, polydatin, penetrates human skin. The stratum corneum of the skin acts as a barrier that only allows small molecules and lipophilic compounds to penetrate [115]. Even cosmetic additives that show efficacy cannot be effective if they cannot penetrate the skin barrier [116]. Although the skin penetrance of polydatin has not been studied, strategies to chemically modify the functional groups of polydatin can be applied to improve the penetration [117]. In addition, the skin penetration of *P. cuspidatum* extract or polydatin can be improved by using carriers. For example, phospholipids or lipid nanoparticles have a structure similar to cell membranes, which can effectively deliver active ingredients that have difficulty penetrating skin cells [118]. Finally, further studies should be conducted to determine whether *P. cuspidatum* extract or polydatin interacts with other ingredients to produce toxic substances. Cosmetic ingredients can interact with each other to form hazardous substances (e.g., nitrosamines) that can have harmful effects on skin [119]. Therefore, intensive studies should be conducted to determine whether hazardous substances are produced when *P. cuspidatum* extract or polydatin is used in various combinations with existing cosmetic ingredients.

## 5. Conclusions

In summary, we discovered a novel mechanism by which *P. cuspidatum* extract reduces ROS levels by increasing mitochondrial OXPHOS efficiency. The reduction in oxidative stress by *P. cuspidatum* extract restored senescence-associated phenotypes and enhanced skin protection. Furthermore, polydatin, one of the components of *P. cuspidatum* extract, was identified as an effective component that exhibits antioxidant activity. Polydatin also increased OXPHOS efficiency and subsequently reduced mitochondrial ROS generation, thereby reducing senescence-associated phenotypes. Our results revealed the novel mechanism of restoring senescence and enhancing skin protection by *P. cuspidatum* extract. If further studies on *P. cuspidatum* extract or polydatin are conducted from various perspectives, the novel mechanism revealed in this study may or may not be applied for clinical or cosmetic purposes in the future, but it will serve as a starting point for various in vitro studies.

## Figures and Tables

**Table 1:** Details of primers used in qPCR.

Target	Orientation	Sequence (5′-3′)	Size (bp)
*p21*	forward	AGGTGGACCTGGAGACTCTCAG	22
	reverse	TCCTCTTGGAGAAGATCAGCCG	22
*IL-1β*	forward	CCACAGACCTTCCAGGAGAATG	22
	reverse	GTGCAGTTCAGTGATCGTACAGG	23
*IL-* *6*	forward	AGACAGCCACTCACCTCTTCAG	22
	reverse	TTCTGCCAGTGCCTCTTTGCTG	22
*Collagen type I*	forward	AGCAAGAACCCCAAGGACAA	20
	reverse	CGAACTGGAATCCATCGGTC	20
*Collagen type* *II* *I*	forward	CTGATGGGGTCAAATGAAGGTG	22
	reverse	CGTGCAACCATCCTCCAGAAC	21
*IL-8*	forward	CTGGCCGTGGCTCTCTTG	18
	reverse	CCTTGGCAAAACTGCACCTT	20
*β* *-defensin 2*	forward	GTATCTCCTCTTCTCGTTCCTC	22
	reverse	GGATCGCCTATACCACCAAAAAC	23

## Data Availability

The original contributions presented in the study are included in the article, further inquiries can be directed to the corresponding authors.

## Supplementary Materials

The following supporting information can be downloaded at https://www.mdpi.com/article/10.3390/antiox13091110/s1, Supplementary Information S1: Full-size image of immunofluorescence in Figure 6A. Supplementary Information S2: Full-size image of western blot (A) Figure 6B, (B) Figure 7A.
