# Supplementary material for: Senescence Rejuvenation through Reduction in Mitochondrial Reactive Oxygen Species Generation by Polygonum cuspidatum Extract: In Vitro Evidence
Source: Antioxidants (Basel). 2024 Sep 14;13(9):1110. doi: 10.3390/antiox13091110 (PMC11429016; doi:10.3390/antiox13091110)
Supplement: Supplementary file 1 [file antioxidants-13-01110-s001.zip › antioxidants-3133397-supplementary.pdf]

Supplementary Information

Figure 6A

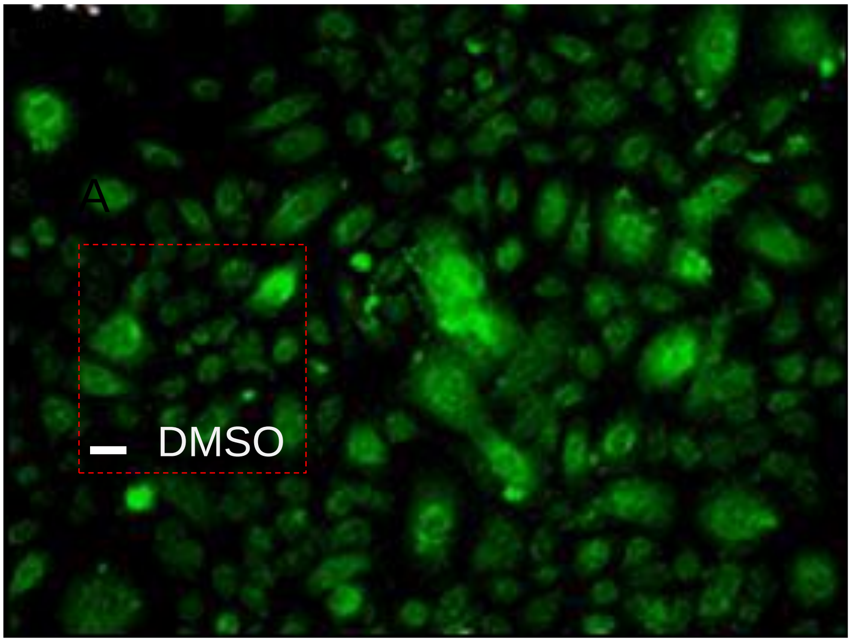

H_2O_2 -

Figure 6A

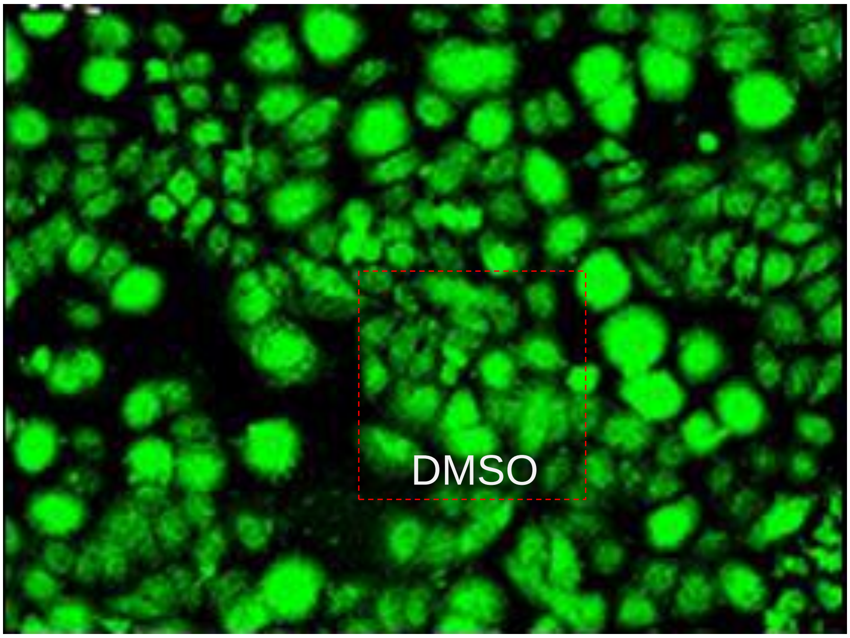

H_2O_2 +

Supplementary Information S1. Full-size image of immunofluorescence in Figure 6A

Supplementary Information

Figure 6A

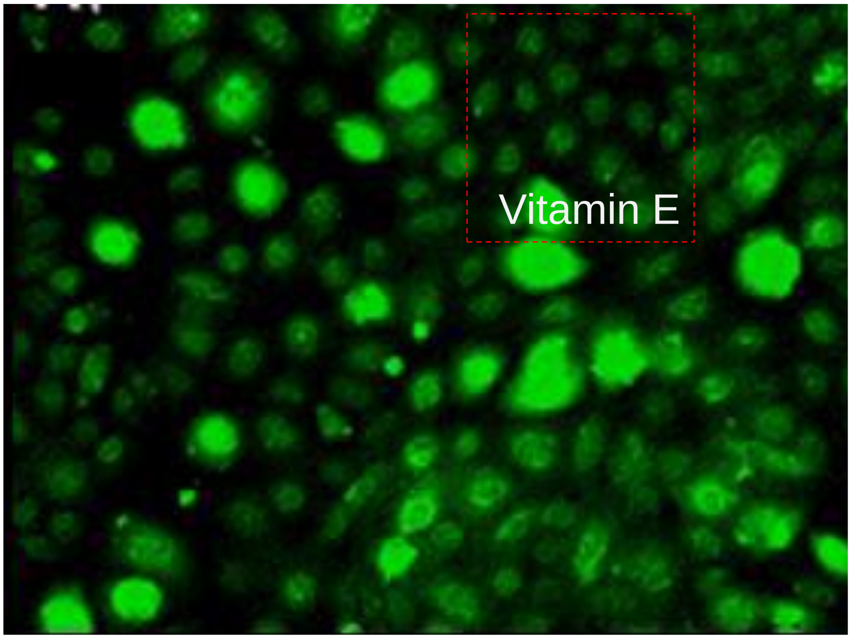

H_2O_2 -

Figure 6A

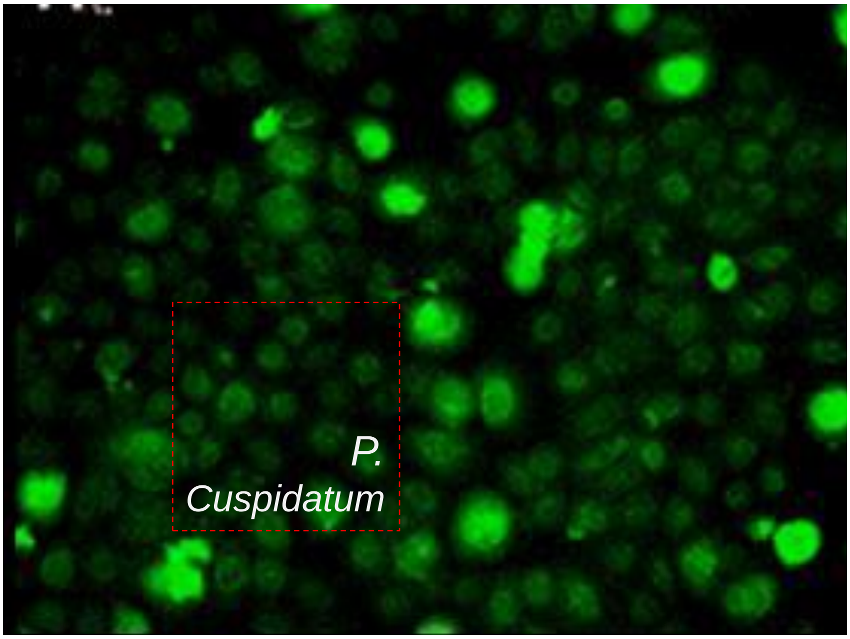

H_2O_2 +

Supplementary Information S1. Full-size image of immunofluorescence in Figure 6A

Supplementary Information

A Figure 6B

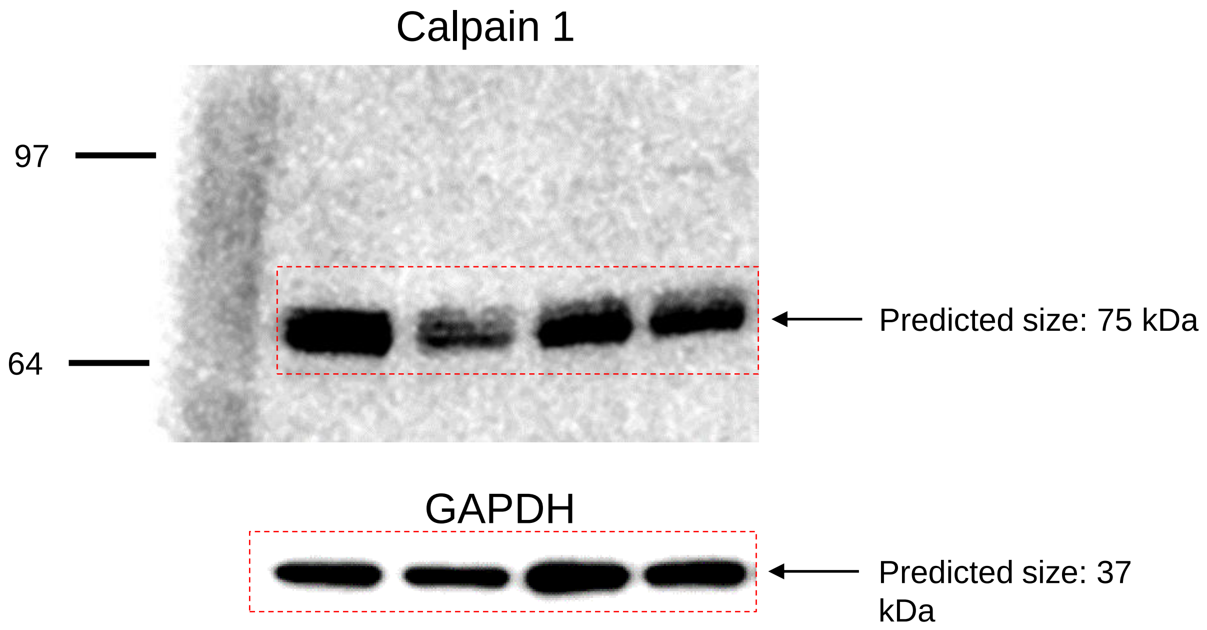

B Figure 7A

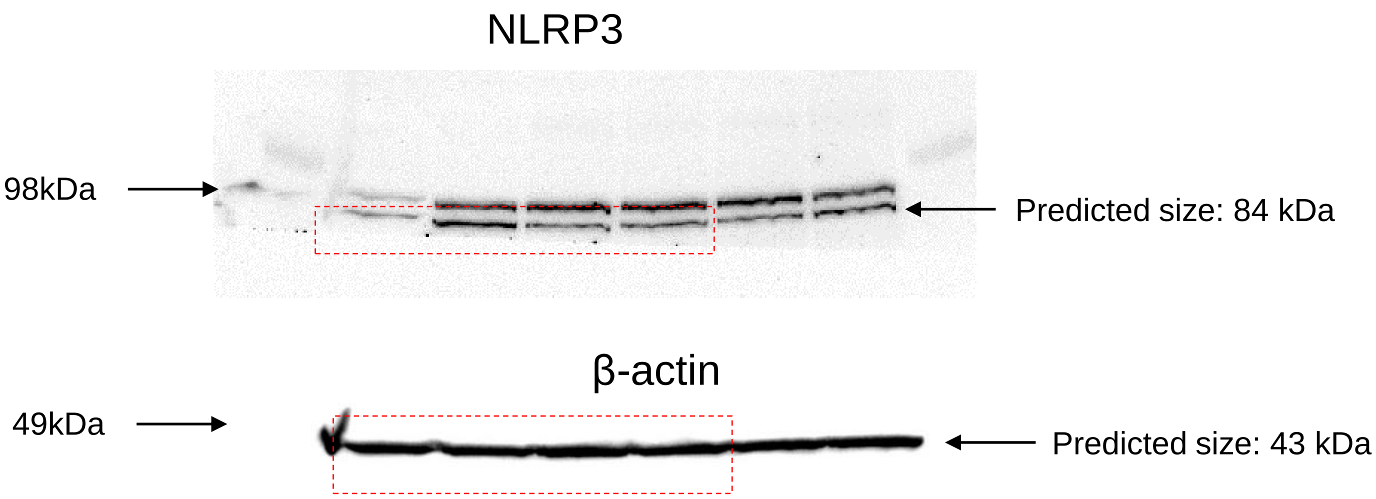

Supplementary Information S2. Full-size image of western blot (A) Figure 6B, **(B)** Figure 7A
